# Supplementary material for: Modeling glioblastoma heterogeneity as a dynamic network of cell states
Source: Mol Syst Biol. 2021 Sep 16;17(9):e10105. doi: 10.15252/msb.202010105 (PMC8444284; doi:10.15252/msb.202010105)
Supplement: Supplementary file 6 — Source Data for Figure 5 [file MSB-17-e10105-s004.zip › Figure5A_sourcedata/GSEA_3017/nosigdb_stateC.GseaPreranked.1622795002785/GANDAL_AUTISM_UP_PMID29439242.html]

Details for gene set GANDAL\_AUTISM\_UP\_PMID29439242[GSEA]

|  || Dataset | state13017 |
| Phenotype | NoPhenotypeAvailable |
| Upregulated in class | na\_neg |
| GeneSet | GANDAL\_AUTISM\_UP\_PMID29439242 |
| Enrichment Score (ES) | -0.585556 |
| Normalized Enrichment Score (NES) | -2.692693 |
| Nominal p-value | 0.0 |
| FDR q-value | 0.0 |
| FWER p-Value | 0.0 |
Table: GSEA Results Summary

  

Fig 1: Enrichment plot: GANDAL\_AUTISM\_UP\_PMID29439242      
 Profile of the Running ES Score & Positions of GeneSet Members on the Rank Ordered List

  

| PROBE | GENE SYMBOL | GENE\_TITLE | RANK IN GENE LIST | RANK METRIC SCORE | RUNNING ES | CORE ENRICHMENT || 1 | HIST1H1C |  |  | 11 | 0.583 | 0.0291 | No |
| 2 | STC1 |  |  | 210 | -0.255 | -0.2953 | No |
| 3 | MYOF |  |  | 251 | -0.263 | -0.3433 | No |
| 4 | DTNA |  |  | 281 | -0.271 | -0.3714 | No |
| 5 | OSMR |  |  | 292 | -0.274 | -0.3662 | No |
| 6 | S100A11 |  |  | 361 | -0.301 | -0.4599 | No |
| 7 | IGFBP5 |  |  | 384 | -0.311 | -0.4725 | No |
| 8 | TIMP1 |  |  | 399 | -0.320 | -0.4704 | No |
| 9 | PDLIM4 |  |  | 466 | -0.370 | -0.5549 | Yes |
| 10 | NQO1 |  |  | 479 | -0.381 | -0.5442 | Yes |
| 11 | IFITM2 |  |  | 483 | -0.388 | -0.5174 | Yes |
| 12 | CEBPD |  |  | 523 | -0.455 | -0.5477 | Yes |
| 13 | IFITM3 |  |  | 533 | -0.477 | -0.5239 | Yes |
| 14 | CRYAB |  |  | 536 | -0.485 | -0.4872 | Yes |
| 15 | COL4A1 |  |  | 545 | -0.507 | -0.4592 | Yes |
| 16 | ANXA2 |  |  | 563 | -0.567 | -0.4418 | Yes |
| 17 | EMP3 |  |  | 568 | -0.580 | -0.4007 | Yes |
| 18 | CD44 |  |  | 575 | -0.659 | -0.3566 | Yes |
| 19 | SPP1 |  |  | 577 | -0.674 | -0.3024 | Yes |
| 20 | TM4SF1 |  |  | 580 | -0.689 | -0.2489 | Yes |
| 21 | S100A10 |  |  | 582 | -0.691 | -0.1933 | Yes |
| 22 | CYR61 |  |  | 584 | -0.714 | -0.1359 | Yes |
| 23 | EMP1 |  |  | 589 | -0.785 | -0.0779 | Yes |
| 24 | CCL2 |  |  | 595 | -1.066 | 0.0017 | Yes |
Table: GSEA details [plain text format]

  

Fig 2: GANDAL\_AUTISM\_UP\_PMID29439242: Random ES distribution      
 Gene set null distribution of ES for **GANDAL\_AUTISM\_UP\_PMID29439242**
